# Supplementary material for: Proximal perimeter encoding in the rat rostral thalamus
Source: Sci Rep. 2019 Feb 27;9:2865. doi: 10.1038/s41598-019-39396-8 (PMC6393499; doi:10.1038/s41598-019-39396-8)

**Supplementary Figure 1**

**Proximal perimeter encoding in the rat rostral thalamus**

Pawel Matulewicz^1,3†^, Katharina Ulrich^1†^, Md Nurul Islam^1^, Mathias L Mathiasen^2^, John P Aggleton^2^, Shane M O'Mara^1*^

^1^Institute of Neuroscience, Trinity College Dublin, Dublin, Ireland; ^2^School of Psychology, Cardiff University, Cardiff, United Kingdom; ^3^Department of Animal and Human Physiology, Faculty of Biology, University of Gdansk, Gdansk, Poland

**Submitted to *Scientific Reports***

*For correspondence: smomara@tcd.ie

^†^These authors contributed equally to this work

**Supplementary Figure 1:** Distribution of the cluster-quality measures or measure of degree of overlap; BC= Bhattacharyya Coefficient, Er= Enclosure ratio, Lr= L-ratio.


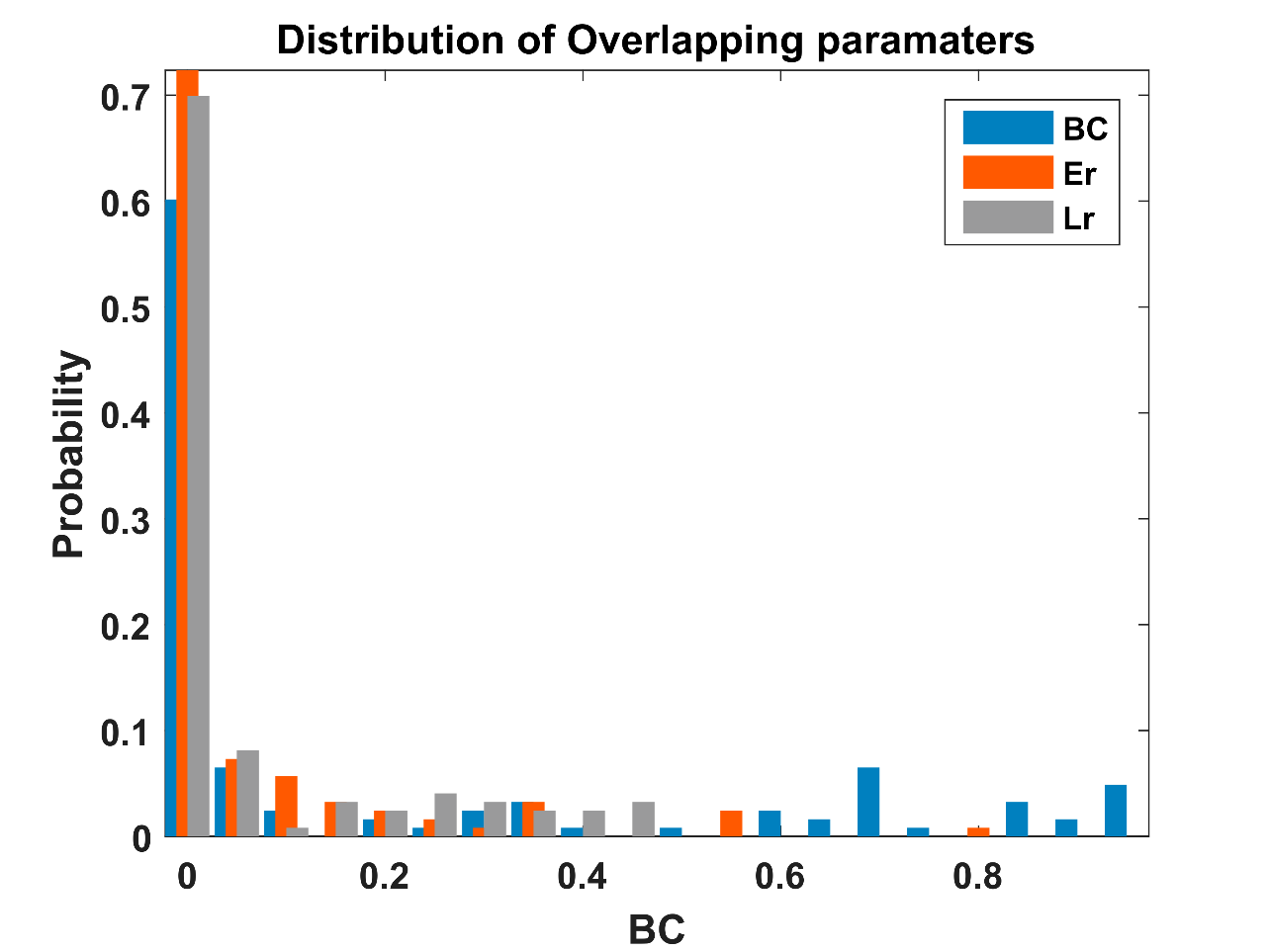

Supplement: Supplementary file 1 — Supplementary Figure 1 [file 41598_2019_39396_MOESM1_ESM.docx]
